# Clinical Spectrum and Genetic Diagnosis of 54 Consecutive Patients Aged 0–25 with Bilateral Cataracts

**DOI:** 10.3390/genes12020131

**Published:** 2021-01-21

**Authors:** Suzannah Bell, Samantha Malka, Ian Christopher Lloyd, Mariya Moosajee

**Affiliations:** 1 Moorfields Eye Hospital NHS Foundation Trust, London EC1V 2PD, UK; suzannah.bell@nhs.net (S.B.); samantha.malka@nhs.net (S.M.); 2 Great Ormond Street Hospital for Children, London WC1N 3JH, UK; ianchristopher.lloyd@gosh.nhs.uk; 3 Manchester Academic Health Sciences Centre, University of Manchester, Manchester M13 9PT, UK; 4 Institute of Ophthalmology, University College London, London EC1V 9EL, UK; 5 The Francis Crick Institute, London NW1 1AT, UK

**Keywords:** genetic testing, targeted gene panels, next-generation sequencing, genomic medicine, childhood/congenital cataracts, retinal dystrophies, aniridia

## Abstract

Childhood cataract affects 2.5–3.5 per 10,000 children in the UK, with a genetic mutation identified in 50–90% of bilateral cases. However, cataracts can also manifest in adolescence and early adulthood in isolation, as part of a complex ocular phenotype or with systemic features making accurate diagnosis more challenging. We investigate our real-world experience through a retrospective review of consecutive bilateral cataract patients (0–25 years) presenting to the ocular genetics service at Moorfields Eye Hospital between 2017 and 2020. Fifty-four patients from 44 unrelated families were identified, with a median age of 13.5 years (range 1 to 68 years) and a median age at diagnosis of 43.9 months IQR (1.7–140.3 months); 40.7% were female and 46.3% were Caucasian. Overall, 37 patients from 27 families (61.4%) were genetically solved (50%) or likely solved (additional 11.4%), with 26 disease-causing variants (8 were novel) in 21 genes; the most common were crystallin genes, in 8 (29.6%) families, with half occurring in the *CRYBB2* gene. There was no significant difference in the molecular diagnostic rates between sporadic and familial inheritance (*P* = 0.287). Associated clinical diagnoses were retinal dystrophies in five (18.5%) and aniridia in three (11.1%) families. Bilateral cataracts were the presenting feature in 27.3% (6/22) of either complex or syndromic cases, and isolated cataract patients were 11.5 years younger (rank-sum Z = 3.668, *P* = 0.0002). Prompt genetic investigation with comprehensive panel testing can aid with diagnosis and optimise management of cataract patients.

## 1. Introduction

Cataract is a leading cause of avoidable visual impairment and blindness in both adults and children worldwide [1,2]. In the UK, cataract affects 2.5–3.5 per 10,000 children, most within the first year of life [3]. It is a highly heterogenous disease with a broad aetiology including congenital infections (*Toxoplasma gondii*, Syphilis, Varicella-Zoster-virus, Parvovirus B19, Coxsackievirus, Rubella, Cytomegalovirus, Herpes Simplex Virus I and II), trauma or radiation, previous ocular surgery or exposure to steroid medications. However, bilateral cases have a genetic preponderance [4,5]. Cataract presenting either at birth or within the first year of life is termed ‘congenital’ and lens opacity presenting later in childhood is described in various ways including infantile, juvenile or developmental cataract. However, hereditary cataracts can also present in later adolescence or early adulthood either in isolation, as part of a complex ocular phenotype (e.g., with retinal dystrophies, anterior segment dysgenesis [ASD], and aniridia) or in association with systemic disorders (syndromic, e.g., cerebrotendinous xanthomatosis [CTX]) [6,7].

Mutations in 115 genes cause cataracts [6]. In isolated cases, mutations occur in genes encoding lens proteins such as crystallins, membrane proteins, cytoskeletal structural proteins and transcription factors that normally maintain lens transparency via their high organised structures, or have a role in homeostasis or lens development [8]. Crystallins are highly refractile proteins making up 80–90% of lens proteins and mutations in these genes are responsible for approximately 50% of non-syndromic cataracts [5,9]. Some genes encoding lens proteins (*CRYBA1, CRYBA2, CRYBA4, CRYBB2, CRYGC, CRYGD, DNMBP, EPHA2, FOXE3, GJA3, GJA8, MAF, NHS, OPA3, P3H2, PAX6, PITX3, PXDN*, and *VSX2*) have a spatiotemporal role, where they regulate the formation of various ocular structures in early eye development, and therefore mutations in these genes cause cataract associated with ocular maldevelopment including ASD, microphthalmia, anophthalmia and ocular coloboma (MAC) [6,7]. Cataracts may also develop in other primary hereditary eye disorders, e.g., in some retinal dystrophies or aniridia. Aniridia is a rare pan-ocular disorder predominately caused by *PAX6* mutations and is typically characterised by congenital, partial or complete iris hypoplasia and foveal hypoplasia with associated nystagmus [10]. It is frequently associated with cataract, glaucoma and limbal stem cell deficiency causing corneal keratopathy [11]. Cataracts are usually mild in infancy but most progress, requiring surgery in the first two decades of life [12]. Posterior subcapsular cataracts are most frequently associated with retinitis pigmentosa (RP). However, the pathophysiology of cataract in this disease is not understood [13]. Aqueous flare values are increased in patients with RP compared to healthy subjects [14], and therefore this may be a risk factor for the development of cataracts, suggesting an inflammatory aetiology [15].

While the aetiology of cataract varies globally, a genetic mutation can be identified in 50–90% of bilateral cases on genetic testing [4,16,17]. While cataracts can be X-linked or autosomal recessive, autosomal dominant cataracts are most common (meaning a 50% risk of passing on the pathogenic variant to any child born to an affected parent). Therefore, genetic testing (and genetic counselling) is a key part of clinical management. However, there is a global lack of access to genetic testing; and in resource-rich settings, a disconnect from general ophthalmology due to externally held department budgets and an uneven distribution of specialist services, with many based in larger cities [18,19]. In the UK, capacity building efforts have already been employed to modify access to genetic testing since the 100,000 Genomes Project, an initiative to sequence the genomes of 85,000 patients with cancer and rare diseases [20]. NHS England plan to extend the use of molecular diagnostics and will routinely offer genomic testing over the next ten years as part of the NHS long-term plan [21,22]. Historically, diagnostic pathways of cataract patients have often been lengthy, inefficient and with poor diagnostic yield [23]. Comprehensive ocular gene panel tests using next-generation sequencing (NGS) and whole-genome sequencing (WGS) have been found to streamline care pathways and alter clinical outcomes for cataract patients in the UK [16,23]. We report our real-world clinical experience of children and young adults presenting with cataracts to the ocular genetics service at Moorfields Eye Hospital NHS Foundation Trust (MEH), which oversees the care of the largest number of genetic eye disease patients of any one site in the United Kingdom, and discuss the impact of genetic testing in this cohort.

## 2. Materials and Methods

Using a search engine of electronic patient records of consecutive patients attending Moorfield Eye Hospital NHS Foundation Trust (MEH) between January 2017 and August 2020, keywords filtered included “juvenile” + “cataract”, “congenital” + “cataract”, “childhood” + “cataract”, “paediatric” + “cataract”, “developmental” + “cataract”, “infantile” + “cataract”, “adolescent” + “cataract”, and “hereditary” + “cataract”. The same search was conducted with “opacity” replacing “cataract”. This search strategy identified 1236 patients, and then OpenEyes (Across Health, Ghent, Belgium) electronic database and the patient’s medical notes were used to retrieve data on demographics, clinical features and genetic results. Patients were eligible for inclusion if they were 0–25 years old at bilateral cataract diagnosis and attended MEH for a review appointment or received a genetic result during the aforementioned timeframe. Molecular testing was performed both in the clinical and research setting using NGS panel testing through the Rare & Inherited Disease Genomic Laboratory at Great Ormond Street Hospital (GOSH) (London, UK) and WGS as part of the UK Genomics England 100,000 Genomes project. Single-gene testing via Sanger sequencing was performed in cases of aniridia (identification of *PAX6* mutation) or for confirmatory testing of research findings (in the case of families 3 and 4); see Table 1. Results were reviewed by a multidisciplinary team (including molecular biologists, clinical geneticists, as well as the ophthalmology specialist managing the family) in order to confirm variant pathogenicity, prevalence in publicly available genome databases, the clinical phenotype and mode of inheritance before the final molecular diagnosis was established. Patients who have characteristic phenotypes that fit variants of unknown significance together with segregation data were considered likely pathogenic. Patients were fully informed about the status of their variants and that it may require further evidence to meet laboratory Association for Clinical Genomic Science (ACGS) requirements, such as another unrelated family with the same mutation and clinical features. The datasets (variants) generated for this study were submitted to ClinVar (https://www.ncbi.nlm.nih.gov/clinvar/) through the Rare & Inherited Disease Genomic Laboratory at GOSH.

Patients were excluded if they had unilateral cataract or cataract due to a known other cause, e.g., trauma, iatrogenic, or inflammatory disease. The date of diagnosis with cataract was unknown for four patients and in these cases, the date of first cataract surgery was used instead (549, 2818, 1826, 6209 days). 

STATA v15 (StataCorp. 2017. Stata Statistical Software: Release 15. College Station, TX: StataCorp LLC) was used for analysis. Age of patient at diagnosis was found to be non-normally distributed and so non-parametric methods were used (Wilcoxon rank-sum test). Chi squared test was used for testing relationships between categorical variables. 

All patients gave written informed consent for genetic testing. This study adhered to the tenets set out in the Declaration of Helsinki and was approved by the London—Camden & Kings Cross Research Ethics Committee (12/LO/0141). Patients tested through the Genomics England 100,000 Genomes project gave written informed consent through REC reference 14/EE/1112, which had relevant local research ethics committee approvals (Moorfields Eye Hospital and the Northwest London Research Ethics Committee).

## 3. Results

**Figure 1:**
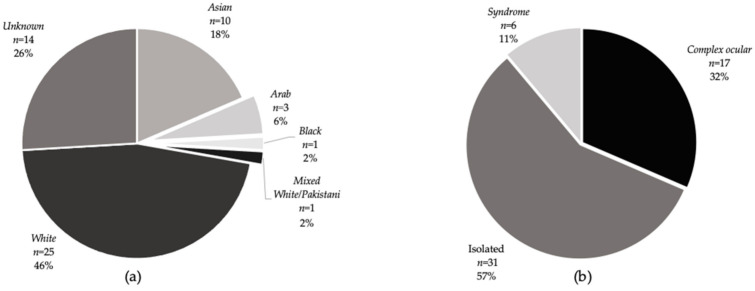
Ethnicities and disease subtypes of individual patients in the cohort. (**a**) Ethnicities of cataract patients as stated on PAS or in the clinical notes. The greatest proportion (25) were of white ethnicity; the ethnicity was not known in 14 patients. (**b**) Proportion of cataract patients by disease subtype. The greatest proportion had an isolated cataract phenotype. Seventeen patients had a complex ocular disease (cataract associated eye pathology) including two with microphthalmia, one with anterior segment dysgenesis as well as patients with retinal dystrophies and aniridia. Six patients had systemic features associated with cataract including one with Stickler syndrome, one with Alstrom syndrome, one with Cerebrotendinous xanthomatosis and three members of family 44 with a *CRYAB* mutation who have systemic features including neuropathy and myopathy in one patient (father) and myopathy only in one patient (daughter) but isolated cataract in one patient (son).

Fifty-four patients from 44 unrelated families (with 44 probands), aged between 1 and 68 years old (median 13.5 years IQR 5–29) presented to the ocular genetics services at MEH. The median age at cataract diagnosis was 43.9 months IQR (1.7–140.3 months), 40.1% of patients were female (22/54) and patient ethnicity (recorded on electronic/hard copy patient records) was White (46.3%, *n* = 25), not stated (25.9%, *n* = 14), Bangladeshi (14.8%, *n* = 8), Arab (5.6%, *n* = 3), Pakistani (1.9%, *n* = 1), Asian, other (1.9%, *n* = 1), Mixed white/Pakistani (1.9%, *n* = 1), and Black African (Nigerian) (1.9%, *n* = 1), (Figure 1a). Patient-reported consanguinity was 6 (21.4% of solved families). Thirty-one families received genetic testing via WGS, 8 received targeted panel sequencing, and 5 received single-gene Sanger sequencing (this was for *PAX6* screening in three patients with aniridia following a previous negative array comparative genomic hybridisation to rule out a deletion involving *WT1* and *PAX6* causing possible WAGR syndrome and two patients as part of research with *OAT* and *CPAMD8* mutation). Two families had received their genetic result elsewhere but had available reports. The genetic results of nine families have been published in an overview of non-retinal developmental eye disorders by our group [31]. Patient demographics including clinical details are listed in Supplementary Materials Table S1.

Overall, 30 patients from 22 families received a molecular result, giving a familial diagnostic rate of 50%. Confirmatory or likely molecular diagnosis, following MDT discussion and expert clinical opinion (as described in Methods) saw this proportion increase to 27/44 (61.4%). Of the 17 families that received no primary finding results, 15 families underwent WGS and 2 had targeted gene panel testing, suggesting that WGS had a diagnostic, or likely diagnostic, rate of 51.6% (16/31 families) and panel testing rate of 75% (6/8 families). Details of genetic results are listed in Table 1. Of these, 13 (48.1%) families had isolated cataract, 10 (37%) had complex ocular cataracts and 4 (14.8%) had a syndromic presentation (Figure 1b). In the complex group, five (18.5%) families had retinal dystrophy, three had aniridia (11.1%), one (3.7%) had anterior segment dysgenesis (*CPAMD8*) and one (3.7%) had microphthalmia with cataracts (*EPHA2*). The confirmed genetic diagnosis in the retinal dystrophy group included RP in three (*AGBL5, RBP3,* and *PRPF8*), Leber congenital amaurosis in one (*RPE65*) and gyrate atrophy in one (*OAT*). From the complex and syndromic cases (*n* = 22), bilateral cataracts were the presenting feature in 27.3% (6/22) patients—two with retinal dystrophies (one unsolved individual and one with *RBP3*-related retinitis pigmentosa 66) and four syndromic cataracts (three individuals with *CRYAB* associated myopathy and one with CTX). Patients with isolated cataracts presented at a younger age (median 191 days IQR (21–1579) than those with complex or syndromic phenotypes (median 12 years IQR (4–23), Wilcoxon rank-sum test Z = 3.668, *P* = 0.0002). There was no difference in the age of presentation between solved and unsolved cases (Z = 0.741, *P* = 0.4584). We identified variants in 16/26 sporadic cases and 21/28 familial cases, which indicated no significant difference in the molecular diagnostic rates between sporadic and familial inheritance (*P* = 0.287). In addition, there was no significant difference (*P* = 0.212) in diagnostic rates between isolated (20/31), complex ocular (11/17) and syndromic cases (6/6).

Posterior subcapsular cataracts were the most frequent cataract type found in 70% of phakic retinal dystrophy patients (7/10). There were no other clear cataract-related genotype-phenotype correlations with significant intra- and inter-familial variability. For example, cataracts (where recorded) varied between and within the four families with *CRYBB2* mutations, including anterior/posterior subcapsular and blue dot, dense central, sutural/blue dot and blue dot (only) cataract types. The three unrelated aniridia patients with *PAX6* variants (patient ID 1-1, 13-1 and 25-1) had posterior subcapsular, posterior cortical and cortical cataracts, respectively. Three individuals had a different cataract type in their left and right eyes; two unsolved patients (patient ID 6-1, 15-1) and the father in family 44 (patient ID 44-1) who had a blue dot cataract in the right and an anterior polar cataract in the left eye (his daughter had bilateral blue dot cataracts and his son was surgically aphakic). 

**Figure 2:**
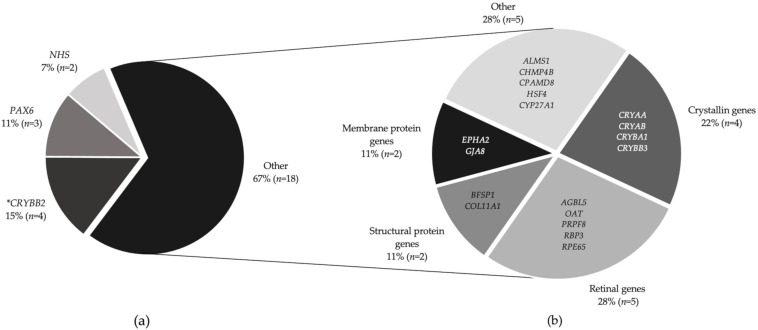
Proportion of families who received a genetic result and their molecular diagnosis. (**a**) Twenty-seven families received a molecular diagnosis. *CRYBB2* mutations were most prevalent (four families), followed by *PAX6* mutations in three families with aniridia and two families had variants in the *NHS* gene. (**b**) Mutations in 18 individual genes affected the remaining 18 families (described as “Other”). * Note *CRYBB2* is also a crystallin gene, hence, together with the other crystallins (*CRYAA, CRYAB, CRYBA1,* and *CRYBB3*), the total contribution is 29.6%.

Most solved families showed an autosomal dominant (AD) pattern of inheritance (66.6%, *n* = 18), then autosomal recessive (25.9%, *n* = 7)) and X-linked (7.4%, *n* = 2) families. Twenty-six disease-causing, or likely disease-causing, variants were found in 21 genes (*AGBL5, ALMS1, BFSP1, CHMP4B, COL11A1, CPAMD8, CRYAA, CRYAB, CRYBA1, CRYBB2, CRYBB3, CYP27A1, EPHA2, GJA8, HSF4, NHS, OAT, PAX6, PRPF8, RBP3*, and *RPE65*) (Figure 2b). Mutations were most frequently associated with crystallin genes, occurring in eight (29.6%) families, with half of those in the *CRYBB2* gene. The most common variant type was missense (51.7%, 15/29), followed by nonsense (20.6%, 6/29), non-coding splice (13.7%, 4/29), frameshift indel (10.3%, 3/29) and small in-frame deletion (3.4%, 1/29). Eight novel variants were discovered in *AGBL5* (c.323C > G p. (Pro108Arg)) *CRYAA* (c.346C > G p. (Arg116Gly)), *CRYBB2*(c.230G > A p. (Gly77Asp)), *CPAMD8* (c.4351T > C p. (Ser1451Pro)), *CYP27A1*(c.1420C > T p. (Arg474Trp)), and *HSF4* c.360+1G > A. Two non-canonical splice mutations in *CRYBA1* (c.215+5G > C) and *BFSP1* (c.957-3C > G) were identified. 

## 4. Discussion

This study has highlighted the real-world clinical spectrum of ocular and systemic conditions that can present with bilateral cataracts in children and young adults. It found that nearly two-thirds of patients were able to receive a molecular diagnosis. Although the majority of cases were isolated, just over 40% of the cohort had other co-morbidities, this points to the need for full investigation so patients receive the correct management and multidisciplinary care. Patients with isolated cataracts were on average 11.5 years younger at diagnosis than those with associated ocular pathology. The majority of our cohort had mutations in crystallin genes, as found in other studies investigating congenital cataracts [9,33]. The Cat-Map database is an online reference database for cataracts in humans and mice. The database shows that approximately 100 mutations in 12 crystallin genes account for half of autosomal dominant cataracts in over 100 families [6,9,33,34].

Cataract types were varied, even within families with the same mutation and individuals. However, most patients in our cohort with retinal dystrophy had posterior subcapsular cataracts, which is a consistent with other studies where inflammatory mechanisms are thought to be involved in cataract development [13,14]. Furthermore, we support previous findings of posterior subcapsular and cortical cataracts in aniridia patients. However, diverse phenotypes can occur in this group likely due to the diverse role of the *PAX6* transcription factor in eye development [35,36].

Cataracts were the presenting feature in over one-quarter of the cohort for non-isolated cases. This suggests that the causative gene may not reside on the cataract-targeted gene panels. For example, in the case of Patient 12-1, who presented with cataracts but was subsequently found to have RP likely to be caused by a mutation in *RBP3* gene, which is not included in routine cataract gene panels. Hence, unsolved individuals must be monitored for emerging clinical signs that may affect the potential differential diagnoses. The different solve rates using WGS and targeted gene panels should also be treated with caution due to our small sample size and varied patient cohort. Other studies have demonstrated variable but similar diagnostic rates in both research and clinical settings using singular testing methods of paediatric bilateral cataract patients only. For example, testing of a 115 cataract-targeted gene panel in 36 bilateral cataract patients by Gillespie et al. determined a genetic aetiology in 75% of cases [16]. However, the same panel used to test 74 children, 5 years or younger with bilateral cataracts, established a genetic diagnosis in 50% of cases [16,17]. The Oculome congenital cataract and lens-associated conditions targeted gene panel with 70 genes established a molecular diagnosis in 88.9% (8/9) patients but the sample size was very small [4]. Findings from a recent real-world study of patients presenting to a single UK ocular genetics service over 12 months with inherited eye diseases found that WGS (through 100,000 Genomes Project) had a diagnostic yield of 44.7% (17/38) for congenital cataract families [31]. 

A variant was identified in 61% of sporadic cases and 75% of familial cases in our cohort but this was not found to be a significant difference. A recent study by Fan et al. of 53 patients with congenital cataracts identified a significant disparity in diagnostic rate between their sporadic (27%) and familial (87.5%) cases (*P* = 0.000) [37]. This difference might be explained by demographic differences in our cohorts. They included monocular cases, which are less likely to have a monogenic cause and are more frequent in their sporadic group, whereas our cohort was older with a higher proportion of patients with complex ocular diseases, e.g., retinal dystrophies. Our higher diagnostic yield in sporadic cases might also be explained by the use of WGS in our cohort.

Recent literature focuses on the clinical utility of genetic testing in rare genetic eye disease to expedite diagnoses and guide optimal patient management, circumventing the need for unnecessary investigations, whilst also leading to cost saving [18,23]. In our cohort, 13-year-old patient 43-I was found to have CTX, a rare but treatable condition, where juvenile cataracts are a frequent, early feature often preceding other neuropsychiatric signs by years. A diagnosis is often not made until adulthood (average 35 years old), by which time, progressive, life-limiting neurological sequelae have occurred [38]. Treatment with daily chenodeoxycholic acid can halt or even reverse neurology if commenced early (studies show effectiveness before 25 years of age), and therefore a genetic diagnosis in this individual will likely improve the patient’s prognosis and avoid significant morbidity and early mortality [39]. Patient 26-I is a 12 year old who presented with developmental delay, nystagmus, photophobia and poor vision in early infancy and was diagnosed at 6 ½ years of age with bilateral cataracts. She was found to have a compound heterozygous *ALMS1* mutation, c.10975C > T p. (Tyr1524*)/ c.4751dup p. (Arg3659*), which causes Alstrom syndrome (OMIM 203800). This autosomal recessive disorder is characterised by progressive cone–rod dystrophy, sensorineural hearing loss, childhood obesity, type 2 diabetes mellitus and dilated cardiomyopathy in 70% of cases. The patient was consequently diagnosed with diabetes and obesity (weight 91st–98th centile, height 25th–50th centile), and she also suffers from autism spectrum disorder but no cardiac or hearing impairments have been detected on regular monitoring by cardiology and audiology. 

Cataract patients encounter numerous barriers to molecular diagnosis because of their varied clinical presentation and broad differential diagnosis. Congenital infections, particularly rubella, are an important cause of cataracts in low-resource settings [40]. However, these are less significant (although still important) in high-resource settings with robust immunisation programmes. Inappropriate use or interpretation of “TORCH” screening tests has the potential for significant clinical consequences for patients and their families [41]. Twenty-eight-year-old patient 19-I had a prenatal history reporting that his mother received serological testing for toxoplasmosis during the third trimester of pregnancy. This led to the assumption that he has maternally derived toxoplasmosis-related congenital cataracts. He sought genetic testing before planning to start a family, which identified an autosomal dominant de novo missense variant in *CRYBB3* (c.466G > A p. (Gly156Arg)), which prompted a family planning referral for pre-implantation genetic diagnosis due to the 50% risk of having an affected child. 

## 5. Conclusions

We expand the mutational spectrum for known cataract genes in young patients. We highlight the extreme heterogeneity of patients presenting with bilateral cataracts and the challenges patients and clinicians face in establishing a molecular diagnosis. We show how a genetic diagnosis can direct individual care pathways, which might include support in family planning and the potential to prevent significant systemic morbidity and mortality in syndromic patients. Despite genetic eye diseases affecting 1 in 1000 people worldwide, ophthalmic genomics is still considered a niche area delivered by a small number of highly trained specialists [18,42]. We highlight the importance of genetic testing in young cataract patients and the benefit of integration of genetic and genomics into UK mainstream ophthalmology.

## Figures and Tables

**Table 1:** Variant details and confirmed phenotype for the 27 solved families presenting to the ocular genetics service in the period 2017–2020. In silico prediction tools used (where relevant): Combined Annotation-Dependent Depletion (CADD) [24]; SpliceAI [25]; PredictSNP2 [26]; SIFT [27]; and PolyPhen-2 [28]. Conservation scores are PhastCons scores (0–1) from 100 vertebrates on Ensembl [29]. Abbreviations: inheritance (Inh.), allele frequency as reported on GnomAD (GnomAD), consanguinity (Cons.), heterozygous variant (Het), compound heterozygous (Compound het), homozygous variant (Hom), hemizygous variant (Hemi), autosomal dominant (AD), autosomal recessive (AR), X-linked recessive (XR). ▲ A novel variant in this gene. ◆ A novel phenotype of microphthalmia was found to associate with cataracts in this family with a dominant heterozygous *EPHA2* variant. * variant is absent from the GnomAD database. ◊ Variant not found in parental samples, so assumed to be de novo, although germline mosaicism has not been excluded. ▪ Both parents shown to carry heterozygous variant.

Family ID	Gene	Disease Name	Zygosity	Transcript	Base Change	Amino Acid Change	Variant Type	Inh.	ACGS Variant Classification	In Silico Prediction Scores	PhastCons Conservation Score	GnomAD	Co-Segregation	Cons.	FH	Family/Patient Reported Before	Variant Prior Reported Phenotype
1	*PAX6*	Aniridia	Het	NM_000280.4	c.2T > G	p. ?	Start codon lost	AD	Pathogenic	CADD 27.7; PredictSNP Neutral (63%)	1	*	N	N	Y	-	Aniridia
2	*COL11A1*	Stickler syndrome, type II	Het	NM_001854.3	c.2755-2A > G▲	-	Non-coding (splice)	AD	Likely Pathogenic	CADD 35; SpliceAI 1.00 (acceptor loss); PredictSNP Deleterious (68%)	1	*	De novo ◊	N	N	-	-
3	*CPAMD8*	Anterior segment dysgenesis 8	Hom	NM_015692.3	c.4351T > C▲	p. (Ser1451Pro)	Missense	AR	VUS	CADD 24.9; PredictSNP Deleterious (87%); PolyPhen-2 Probably damaging (1.000)	1	*	Y ▪	Y	N	-	-
4	*OAT*	Gyrate atrophy of choroid and retina	Compound het	NM_000274.4	c.596C > A; c.1250C > T	p. Pro199Gln; p. Pro417Leu	Missense	AR	Pathogenic; Pathogenic	CADD 27.1 / 29.0; PredictSNP Deleterious (82% / 87%); PolyPhen-2 Probably damaging (0.992 / 1.000)	1; 1	1.414 × 10^−5^; 2.833 × 10^−5^	N	N	N	-	Gyrate Atrophy
5	*EPHA2* *◆*	Cataract 6, multiple types	Het	NM_004431.3	c.1751C > T	p. Pro584Leu	Missense	AD	Likely Pathogenic	CADD 25.3; PredictSNP Deleterious (87%); PolyPhen-2 Probably damaging (0.997)	1	*	Y—variant in two affected relatives	N	Y	[28]	Congenital cataract
7	*AGBL5*	Retinitis pigmentosa 75	Hom	NM_021831.5	c.323C > G▲	p. Pro108Arg	Missense	AR	Likely Pathogenic	CADD 25.7; PredictSNP Deleterious (87%); PolyPhen-2 Probably damaging (1.000)	1	*	Y ▪	Y	Sibling only	[30]	-
8	*RPE65*	Leber congenital amaurosis 2	Hom	NM_000329.3	c.1067dupA	p. Asn356LysfsTer9	Frameshift (duplication)	AR	Pathogenic	PredictSNP; SIFT Damaging (0.858)	1	*	Y ▪	Y	N	-	Leber congenital amaurosis 2
9	*CRYBB2*	Cataract 3, multiple types	Het	NM_000496.2	c.230G > A▲	p. (Gly77Asp)	Missense	AD	VUS	CADD 27.3; PredictSNP Deleterious (87%); PolyPhen-2 Probably damaging (1.000)	1	*	Y—variant in affected mother	N	Y	-	-
10	*HSF4*	Cataract 5, multiple types	Het	NM_001040667.2	c.360+1G > A▲	-	Missense	AD	Likely Pathogenic	CADD 35; SpliceAI 0.98 (donor loss); PredictSNP Deleterious (89%)	1	*	Y—variant in affected father	N	Y	[31]	-
12	*RBP3*	Retinitis pigmentosa 66	Hom	NM_002900.2	c.832_834delTTC	p. (Phe278del)	In-frame deletion	AR	VUS	PredictSNP; SIFT Damaging (0.894)	1	2.173 × 10^−5^	N	N	N	-	-
13	*PAX6*	Aniridia	Het	NM_000280.4	c.718C > T	p. Arg240Ter	Nonsense	AD	Likely Pathogenic	CADD 37; PredictSNP Deleterious (79%)	1	*	N	N	N	-	Aniridia
16	*CRYAA*	Cataract 9, multiple types	Het	NM_000394.2	c.346C > G▲	p. (Arg116Gly)	Missense	AD	Likely Pathogenic	CADD 28.4; PredictSNP Deleterious (87%); PolyPhen-2 Probably damaging (1.000)	1	*	Y—variant in 3+ affected family members	N	Y	-	-
19	*CRYBB3*	Cataract 22	Het	NM_004076.4	c.466G > A	p. (Gly156Arg)	Missense	AD	Likely Pathogenic	CADD 27.3; PredictSNP Deleterious (87%); PolyPhen-2 Probably damaging (1.000)	1	3.19 × 10^−5^	N	N	N	[31]	Congenital cataract
22	*CRYBB2*	Cataract 3, multiple types	Het	NM_000496.2	c.355G > A	p. (Gly119Arg)	Missense	AD	Likely Pathogenic	CADD 29.6; PredictSNP Deleterious (81%); PolyPhen-2 Probably damaging (1.000)	1	*	De novo ◊	N	N	[31]	Cataract 3
23	*BFSP1*	Cataract 33, multiple types	Het	NM_001195.4	c.957-3C > G▲	-	Non-coding (splice)	AD	VUS	CADD 24.6; SpliceAI 0.76 (acceptor loss); PredictSNP Deleterious (97%)	0.992	*	Y—variant in affected father	N	Y	-	-
24	*PRPF8*	Retinitis pigmentosa 13	Het	NM_006445.3	c.5804G > A	p. (Arg1935His)	Missense	AD	Likely Pathogenic	CADD 28.3; PredictSNP Deleterious (87%); PolyPhen-2 Probably damaging (1.000)	1	*	De novo ◊	N	N	-	Retinitis Pigmentosa
25	*PAX6*	Aniridia	Het	NM_000280.4	c.1184-1G > C	-	Non-coding (splice)	AD	Pathogenic	CADD 34; SpliceAI 0.97 (acceptor loss); PredictSNP Deleterious (89%)	1	*	N	N	Y	-	-
26	*ALMS1*	Alstrom syndrome	Compound het	NM_015120.4	c.4569dup; c.10975C > T	p. (Tyr1524LeufsTer5); p. (Arg3659Ter)	Nonsense; Frameshift (duplication)	AR	Pathogenic; Pathogenic	Frameshift—SIFT Damaging (0.858); Nonsense—PredictSNP2 Neutral (60%)	0; 0	*; 4.02 × 10^−6^	Y—variants in trans	N	N	-	Alstrom syndrome
27	*NHS*	Cataract 40, X-linked	Hemi	NM_198270.3	c.245dup	p. (Pro83AlafsTer100)	Nonsense	XR	Pathogenic	PredictSNP ; SIFT Damaging (0.579)	0.945	*	N	N	Y	[31]	-
28	*CRYBB2*	Cataract 3, multiple types	Het	NM_000496.2	c.463C > T	p. (Gln155Ter)	Nonsense	AD	Pathogenic	CADD 45; PredictSNP N/A (low confidence)	1	*	Y—variant in 4 affected relatives	N	Y	[31]	Congenital cataract
34	*GJA8*	Cataract 1, multiple types	Het	NM_005267.4	c.134G > T	p. (Trp45Leu)	Missense	AD	Likely Pathogenic	CADD 28.2; PredictSNP Deleterious (82%); PolyPhen-2 Probably damaging (1.000)	1	*	De novo ◊	N	N	[31]	Cataract
37	*NHS*	Cataract 40, X-linked	Hemi	NM_001291867.2	c.1625del	p. Pro542LeufsTer35	Nonsense	XR	Likely Pathogenic	PredictSNP; SIFT Damaging (0.858)	0.979	*	Y—unaffected mother is carrier	N	N	-	-
40	*CRYBB2*	Cataract 3, multiple types	Het	NM_000496.2	c.355G > A	p. (Gly119Arg)	Missense	AD	Likely Pathogenic	CADD 29.6; PredictSNP Deleterious (81%); PolyPhen-2 Probably damaging (1.000)	1	*	Y—variant absent in unaffected mother, present in affected sister	N	Y	-	Cataract 3
41	*CHMP4B*	Cataract 31, multiple types	Het	NM_176812.4	c.481G > C	p. (Glu161Gln)	Frameshift deletion	AD	Likely Pathogenic	CADD 33; PredictSNP Deleterious (87%); PolyPhen-2 Probably damaging (1.000)	1	*	Y—variant in affected father	N	Y	[31,32]	Cataract 31
42	*CRYBA1*	Cataract 10, multiple types	Het	NM_005208.4	c.215+5G > C▲	-	Non-coding (splice)	AD	VUS	CADD 27.9; SpliceAI 0.98 (donor loss); PredictSNP Deleterious (97%)	1	*	Y—variant in unaffected mother (non-penetrance—maternal relatives affected)	N	Y	-	-
43	*CYP27A1*	Cerebrotendinous xanthomatosis	Hom	NM_000784.3	c.1420C > T▲	p. (Arg474Trp)	Missense	AR	Likely Pathogenic	CADD 29.4; PredictSNP Deleterious (87%); PolyPhen-2 Probably damaging (1.000)	0.984	2.396 × 10^−5^	N	Y	N	-	Cerebrotendinous xanthomatosis
44	*CRYAB*	Cataract 16, multiple type	Het	NM_001885.2	c.358A > G	p. (Arg120Gly)	Missense	AD	Pathogenic	CADD 26.7; PredictSNP Deleterious (87%); PolyPhen-2 Probably damaging (1.000)	1	*	Y—variant in 2 affected relatives	N	Y	-	Myofibrillar Myopathy

## Data Availability

Data are available upon request.

## Supplementary Materials

The following are available online at https://www.mdpi.com/2073-4425/12/2/131/s1, Table S1. Demographic details for the 54 individual patients (solved/unsolved) and their clinical features including cataract type, details of ocular co-morbidities, systemic features and whether cataract was the presenting feature (non-isolated cataract patients only). Abbreviations: yes (Y), no (N), not applicable (N/A).
