# Supplementary material for: Clinical Spectrum and Genetic Diagnosis of 54 Consecutive Patients Aged 0–25 with Bilateral Cataracts
Source: Genes (Basel). 2021 Jan 21;12(2):131. doi: 10.3390/genes12020131 (PMC7909819; doi:10.3390/genes12020131)
Supplement: Supplementary file 1 [file genes-12-00131-s001.pdf]

Supplementary Materials:

Supplementary table 1. Demographic details for the 54 individual patients (solved/unsolved) and their clinical features including cataract type, details of ocular co-morbidities, systemic features and whether cataract was the presenting feature (non-isolated cataract patients only). Abbreviations: yes (Y), no (N), not applicable (N/A).

Patient ID	Family ID	Gene	Confirmed genetic diagnosis	Ethnicity	Age	M/F	Age at diagnosis (days)	Age at surgery	Cataract type RE	Cataract type LE	Ocular co-morbidities	Cataract presenting sign	Systemic features	Consanguinity	FH
**1-1**	1	PAX6	Aniridia	White British	25	F	23 years 4 months	-	Posterior subcapsular	Posterior subcapsular	Aniridia, nystagmus, glaucoma, foveal hypoplasia	N	N	N	Y
**2-1**	2	COL11A1	Stickler syndrome, type II	Not Stated	34	F	7 years 9 months	7 years 8 months	Aphakia (post-surgical)	Aphakia (post-surgical)	Macular atrophy, myopia, lens subluxation, vitreous anomaly	N	Cleft palate, epilepsy, high arched palate, flattened maxilla, short stature (5'2ft)	N	N
**3-1**	3	CPAMD8	Anterior segment dysgenesis 8	Other, Any other	27	F	12 years 5 months	-	Posterior subcapsular	Posterior subcapsular	Anterior segment dysgenesis, pupillary abnormalities including ectopic pupils, ectropion UVAE and irodensis, nystagmus, dysplastic optic discs, large corneal diameters	N	N	Y	N
**4-1**	4	OAT	Gyrate atrophy of choroid and retina	White British	42	F	23 years 7 months	29 years 1 month	Posterior subcapsular	Posterior subcapsular	Retinal dystrophy, exotropia	N	Bipolar disorder	N	N
**5-1**	5	EPHA2	Cataract 6, multiple types	White British	43	F	1 year 6 months	1 year 6 months	Pseudophakia	Pseudophakia	Microcornea, nystagmus, esotropia	Y with microphthalmia	Migraine	N	Y
**5-2**	5	EPHA2	Cataract 6, multiple types	White British	5	M	1 month	1 month	Aphakia (post-surgical)	Aphakia (post-surgical)	Microcornea, micropthalma nystagmus	Y with microphthalmia	N	N	Y
**6-1**	6	Negative/unsolved	-	White, Any other	26	M	20 years 6 months	-	Posterior subcapsular	Dense	Retinal dystrophy, optic atrophy, epiretinal membrane	N	Developmental delay, benign essential tremor(normal MRI), sensorineural hearing loss	N	N

**7-1**	7	AGBL5	Retinitis pigmentosa 75	White, Any other	31	F	23 years 9 months	24 years 1 month	Posterior subcapsular	Posterior subcapsular	Retinal dystrophy, macular oedema	N	Learning difficulties, obesity	Y	Sibling only
**8-1**	8	RPE65	Leber congenital amaurosis 2	Asian Bangladeshi	13	M	12 years 5 months	-	Blue dot	Blue dot	Retinal dystrophy, nystagmus	No	No	Y	N
**9-1**	9	CRYBB2	Cataract 3, multiple types	White British	12	F	<1 month (21 days)	2 years 8 months	"congenital"	"congenital"	Myopia	N/A	N	N	Y
**9-2**	9	CRYBB2	Cataract 3, multiple types	White British	4	M	<1 month (25 days)	3 months	"congenital"	"congenital"	N	N/A	N	N	Y
**10-1**	10	HSF4	Cataract 5, multiple types	Other, any other	2	M	2 months	2 months	"congenital"	"congenital"	N	N/A	N	N	Y
**11-1**	11	Negative/unsolved	-	Other, Any other	4	M	1 year 5 months	1 year 8 months	Nuclear	Nuclear	Anisometropia	N/A	N	Y	Y
**12-1**	12	RBP3	Retinitis pigmentosa 66	Not Stated	29	M	17 years	17 years	Pseudophakia	Pseudophakia	Retinal dystrophy, myopia	Yes	No	N	N
**13-1**	13	PAX6	Aniridia	White British	8	F	3 months	-	Posterior cortical	Posterior cortical	Aniridia, nystagmus, persistent pupillary membrane, foveal hypoplasia,	Yes with aniridia	No	N	N
**14-1**	14	Negative/unsolved	-	White, Any other	29	F	23 years 6 months	27 years	Posterior subcapsular	Posterior subcapsular	Myopia, nystagmus, exudative vitreoretinopathy, vitreous haemorrhage, optic nerve head myelination, reitnal detachment	N	N	N	N
**15-1**	15	Negative/unsolved	-	Asian Bangladeshi	15	M	10 years 4 months	13 years 2 months	Nuclear	Blue dot	High myopia, viteroretinopathy	N	Obesity	N	N
**16-1**	16	CRYAA	Cataract 9, multiple types	White British	5	F	< 1 month (12 days)	3 months	"congenital"	"congenital"	N	N/A	N	N	Y

**17-1**	17	Negative/unsolved	-	White British	24	M	8 years	-	Lamellar	Lamellar	Left superior oblique muscle palsy	N/A	N	N	N
**18-1**	18	Negative/unsolved	-	White British	20	M	At birth	1 month	Aphakia (post-surgical)	Aphakia (post-surgical)	N	N/A	N	N	Sibling only
**19-1**	19	CRYBB3	Cataract 22	Other, any other	32	M	2 days	3 months	Aphakia	Aphakia	Nystagmus	N/A	N	N	N
**20-1**	20	Negative/unsolved	-	White British	29	M	5 years	4 years	Pseudophakia	Pseudophakia	Macular dystrophy, band keratopathy, esotropia	N/A	Myeloproliferative disorder (splenectomy and liver transplant)	N	N
**21-1**	21	Negative/unsolved	-	White British	15	M	3 years 7 months	-	Lamellar pulverulent	Lamellar pulverulent	Myopia, esotropia	N/A	Migraine	N	N
**22-1**	22	CRYBB2	Cataract 3, multiple types	White, British	7	M	< 1 month (13 days)	1 month	"Dense central"	"Dense central"	N	N/A	N	N	N
**23-1**	23	BFSP1	Cataract 33, multiple types	White, British	46	M	2 years	27 years 7 months	Pseudophakia	Pseudophakia	High myopia	N/A	N	N	Sibling only
**23-2**	23	BFSP1	Cataract 33, multiple types	White, British	14	M	4 years 4 months	4 years 4 months	"congenital"	"congenital"	N	N/A	N	N	Sibling only
**24-1**	24	PRPF8	Retinitis pigmentosa 13	Arab	17	F	15 years 6 months	16 years 8 months	Posterior subcapsular	Posterior subcapsular	Retinal dystrophy	No	No	N	N
**25-1**	25	PAX6	Aniridia	Other, Any other	31	M	24 years 10 months	-	Cortical	Cortical	Aniridia, foveal hypoplasia, nystagmus	Yes with aniridia	No	N	Y
**26-1**	26	ALMS1	Alstrom syndrome	Black African (Nigerian)	11	F	6 years 6 months	6 years 6 months	Anterior subcapsular	Anterior subcapsular	Retinal dystrophy, nystagmus, achromatopsia, mild hypermetropia, exotropia	N	Asthma, autism, developmental delay, learning difficulties, diabetes mellitis, obesity, acanthosis nigracans.	N	N

													Normal height (25th-50th centile). Normal cardiac function.		
**27-1**	27	NHS	Cataract 40, X-linked	White, British	68	M	2 years	5 years 7 months	Aphakia	Aphakia	Nystagmus	N/A	Marfan syndrome	N	Y
**28-1**	28	CRYBB2	Cataract 3, multiple types	Asian Bangladeshi	34	M	20 years	-	Aphakia	Aphakia	N	N/A	N	N	Y
**28-2**	28	CRYBB2	Cataract 3, multiple types	Asian Bangladeshi	12	M	10 years	-	Anterior/posterior subcapsular and blue dot	Anterior/posterior subcapsular and blue dot	Myopic astigmatism	N/A	N	N	Y
**28-3**	28	CRYBB2	Cataract 3, multiple types	Asian Bangladeshi	10	M	7 years 11 months	-	Sutural/blue dot	Sutural/blue dot	Myopic astigmatism	N/A	N	N	Y
**28-4**	28	CRYBB2	Cataract 3, multiple types	Asian Bangladeshi	7	F	4 years 4 months	21 years	Blue dot	Blue dot	Myopic astigmatism	N/A	N	N	Y
**29-1**	29	Negative/unsolved	-	Not Stated	6	M	4 years	6 years 2 months	Posterior subcapsular	Posterior subcapsular	Retinal dystrophy	Yes with retinal changes	N	Y	N
**30-1**	30	Negative/unsolved	-	Asian Bangladeshi	3	M	8 months	10 months	"congenital"	"congenital"	Nystagmus	Yes	No	N	Y
**31-1**	31	Negative/unsolved	-	White British	2	M	2 months	3 months	Blue dot	Blue dot	Nystagmus, esotropia	N/A	Developmental delay	N	N
**32-1**	32	Negative/unsolved	-	White British	15	M	14 years	16 years 8 months	Blue dot	Blue dot	N	N/A	ADHD	N	N
**33-1**	33	Negative/unsolved	-	Mixed White/Pakistani	4	F	2 years 4 months	2 years 7 months	Cortical/lenticular	Cortical/lenticular	Exotropia, myopia,	N/A	N	N	Y

**34-1**	34	GJA8	Cataract 1, multiple types	White British	3	F	2 months	2 months	"congenital"	"congenital"	N	N/A	N	N	N
**35-1**	35	Negative/u nsolved	-	Other, Any other	2	F	4 months	-	Anterior polar	Anterior polar	N	N/A	N	N	N
**36-1**	36	Negative/u nsolved	-	Asian, Pakistani	39	M	23 years	36 years 7 months	Posterior subcapsular	Posterior subcapsular	Retinal detachment, retinal dystrophy, macular atrophy	N	Type 1 diabetes, deafness, renal failure. No cardiac manifestations .	N	N
**37-1**	37	NHS	Cataract 40, X-linked	Other, any other	1	M	2 months	2 months	Nuclear	Nuclear	N	N/A	N	N	N
**38-1**	38	Negative/u nsolved	-	White British	2	F	< 1 month (7 days)	-	Anterior polar	Anterior polar	Anisometropia	N/A	N	N	N
**39-1**	39	Negative/u nsolved	-	Not Stated	12	M	11 years 5 months	-	Posterior subcapsular / blue dot	Posterior subcapsular / blue dot	Generalised rod dysfunction	Y	Ehlers Danlos syndrome, severe asthma, eczema, multiple food allergies, eosinophillic oesophagitis	N	N
**40-1**	40	CRYBB2	Cataract 3, multiple types	Arab	6	F	At birth	2 months	Aphakia (post-surgical)	Aphakia (post-surgical)	Blepharitis, esotropia	N/A	Asthma, allergy (sweetcorn, nuts, eggs)	N	Y
**40-2**	40	CRYBB2	Cataract 3, multiple types	Arab	1	F	At birth	2 months	"congenital"	"congenital"	N	N/A	N	N	Y
**41-1**	41	CHMP4B	Cataract 31, multiple types	White, Any other	55	M	Unknow n	-	Aphakia	Aphakia	N	N/A	N	N	Y
**41-2**	41	CHMP4B	Cataract 31, multiple types	White, Any other	4	F	3 years 7 months	3 years 8 months	Posterior subcapsular	Posterior subcapsular	N	N/A	N	N	Y
**42-1**	42	CRYBA1	Cataract 10, multiple types	Asian, Any other	1	M	4 months	-	Posterior subcapsular	Posterior subcapsular	N	N/A	N	N	Y

**43-1**	43	CYP27A1	Cerebrotendinous xanthomatosis	Asian Bangladeshi	13	F	11 years 8 months	-	Blue dot	Blue dot	Myopic astigmatism	Y	Developmental delay, intellectual disability	Y	N
**44-1**	44	CRYAB	Cataract 16, multiple types	Not stated	62	M	Unknown	-	Blue dot	Anterior polar	N	Y	Neuropathy, myopathy, diaphragmatic weakness and hypertension	N	Y
**44-2**	44	CRYAB	Cataract 16, multiple types	Not stated	30	M	At birth	-	Aphakia	Aphakia	N	Y	N	N	Y
**44-3**	44	CRYAB	Cataract 16, multiple types	Not stated	28	F	< 1 month (3 weeks)	4 months	Sutural/blue dot	Sutural/blue dot	N	Y	Muscular weakness	N	Y
